# Building and Using Data Resources for Research on Job Characteristics, Health, and Retirement

**DOI:** 10.1093/geroni/igab046.885

**Published:** 2021-12-17

**Authors:** Amanda Sonnega, Gwen Fisher

## Abstract

A growing literature seeks to understand the relationship between the experience of work and important later-life outcomes. Rich longitudinal measurement of both sides of this equation in datasets such as the Health and Retirement Study (HRS) have made this research possible. These data take the form of self-reported experiences of work (such as physical demands, job flexibility, job satisfaction etc.). Increasingly, researchers are looking to add potentially complementary information on the work environment available in the Occupational Information Network (O*NET) database through a linkage using occupation and industry codes in the survey data. The session talks will describe research conducted using O*NET linked with HRS data as well as ongoing work to create a new data resource that will allow other researchers to undertake research with O*NET-HRS linked data. Each presentation will include some discussion of both the value and limits of using the linkage to O*NET. Carpenter will provide a detailed description a new project linking the 2019 O*NET data to the HRS for public use.This presentation explains the types of variables that will be made available in the O*NET-HRS occupation project and will provide examples for how the measures can be used in longitudinal HRS studies. Using O*NET-HRS linked data, Carr will present on work examining the role of preretirement job complexity in alternative retirement paths and cognitive performance. Helppie-McFall will used the linked data to discuss the role of mismatch between demands of work and workers’ ability to meet those demands in retirement decisions.

